# Sigma factor 1 in chloroplast gene transcription and photosynthetic light acclimation

**DOI:** 10.1093/jxb/erz464

**Published:** 2019-10-23

**Authors:** Lauren A Macadlo, Iskander M Ibrahim, Sujith Puthiyaveetil

**Affiliations:** 1 Department of Biochemistry and Purdue Center for Plant Biology, Purdue University, West Lafayette, IN 47906, USA; 2 Australian National University, Australia

**Keywords:** Chloroplast gene transcription, light acclimation, photosynthesis, photosystem stoichiometry adjustment, phytochrome, redox signaling, sigma factor 1, transcriptional control

## Abstract

Sigma factors are dissociable subunits of bacterial RNA polymerase that ensure efficient transcription initiation from gene promoters. Owing to their prokaryotic origin, chloroplasts possess a typical bacterial RNA polymerase together with its sigma factor subunit. The higher plant *Arabidopsis thaliana* contain as many as six sigma factors for the hundred or so of its chloroplast genes. The role of this relatively large number of transcription initiation factors for the miniature chloroplast genome, however, is not fully understood. Using two Arabidopsis T-DNA insertion mutants, we show that sigma factor 1 (SIG1) initiates transcription of a specific subset of chloroplast genes. We further show that the photosynthetic control of PSI reaction center gene transcription requires complementary regulation of the nuclear *SIG1* gene at the transcriptional level. This *SIG1* gene regulation is dependent on both a plastid redox signal and a light signal transduced by the phytochrome photoreceptor.

## Teaser

Sigma factor 1 initiates transcription of a specific subset of chloroplast genes in Arabidopsis. Photosynthetic control of *psaAB* transcription requires a light quality-mediated regulation of the nuclear sigma factor 1 gene.

## Introduction

Sigma factors are subunits of bacterial RNA polymerases. They enable efficient transcription of bacterial genes by their three distinct activities: by imparting a promoter recognition property to the RNA polymerase; by melting the double-stranded promoter regions into transcription-competent, single-stranded open complexes; and by interacting with other DNA-binding transcription factors for regulated gene expression (Paget and Helmann, 2003; Davis *et al.*, 2017). Chloroplasts are cytoplasmic organelles in which photosynthesis takes place in plants and algae. By virtue of their cyanobacterial ancestry, chloroplasts contain a small transcriptionally active genome and a bacterial gene expression machinery (Keeling, 2010). The chloroplast genome typically contains 100–300 genes, which are mostly organized in polycistronic operons as in bacteria. Most chloroplast genes have bacterial-type –10 and –35 promoter elements, which are recognized and transcribed by a multisubunit eubacterial RNA polymerase (Weihe and Börner, 1999). The core subunits of this bacterial-type polymerase are encoded in the plastid genome and hence named as the plastid-encoded polymerase (PEP). Like the bacterial polymerase, the core PEP enzyme requires the reversibly binding, nuclear-encoded sigma factor subunit for efficient transcription. In flowering plants and moss, one or more single-subunit phage-type RNA polymerases, known as the nuclear-encoded polymerases (NEPs), transcribe a small subset of chloroplast genes from distinct promoter elements (Börner *et al.*, 2015). These NEP-transcribed genes include *rpoB*, encoding the β-subunit of PEP, and a few tRNA genes. Some chloroplast genes contain both PEP and NEP promoters and are transcribed by both polymerases in all stages of plastid development and all plant tissue types (Börner *et al.*, 2015).

The chloroplast proteome contains ~3000 proteins, of which only <5% are encoded in the chloroplast genome (Martin *et al.*, 2002). The rest are products of nuclear genes. The diminutive chloroplast genome is nevertheless over-represented by genes that encode core subunits of the photosynthetic electron transport complexes (Allen *et al.*, 2011). Two large pigment protein complexes, PSII and PSI, form the functional units of photosynthesis. They undertake the light harvesting and the primary light-driven electron transport reactions of photosynthesis. A cytochrome *b*_6_*f* complex (Cyt *b*_6_*f*) connects the two photosystems in series. These three protein complexes together carry out the linear electron transport from water to NADP^+^, generating the electron-rich NADPH molecule and a proton motive force that drives the synthesis of ATP through the ATP synthase enzyme. The NAD(P)H dehydrogenase-like complex (NDH), a homolog of respiratory complex I, forms a further electron transport complex in chloroplasts. The function of NDH is the cycling of electrons around PSI in order to build a proton motive force that drives the synthesis of additional ATP molecules (Shikanai, 2016; Strand *et al.*, 2017; Laughlin *et al.*, 2019). The core protein subunits of all five complexes are products of chloroplast genes. Some of these chloroplast-encoded subunits act as dominant assembly factors for *de novo* complex biogenesis and thus set the pace of complex formation in the photosynthetic thylakoid membrane (Choquet and Vallon, 2000; Nickelsen and Rengstl, 2013). Regulation of the gene expression of these dominant assembly factors could therefore adjust the relative abundance of electron transport complexes, optimizing electron flux and photosynthetic efficiency in changing light conditions (Pfannschmidt *et al.*, 1999).

Chloroplast sigma factors belong to the large sigma 70 family of sigma factors (Chi *et al.*, 2015). The model higher plant *Arabidopsis thaliana* contain as many as six chloroplast sigma factors (Schweer *et al.*, 2010*a*). Biochemical and molecular genetic studies reveal a certain degree of functional specialization among chloroplast sigma factors, with each seemingly possessing a unique set of target genes that are transcribed in an environment- and plant development-dependent fashion (Lerbs-Mache, 2011; Yagi and Shiina, 2014; Chi *et al.*, 2015). Sigma factor 1 (SIG1) appears to be a major housekeeping sigma factor in plant chloroplasts, but its target genes are not yet fully identified in Arabidopsis (Chi *et al.*, 2015). The recombinant Arabidopsis SIG1 binds to *psbA* and *rbcL* gene promoters *in vitro*, albeit with a lower affinity (Privat *et al.*, 2003). In rice, knocking out the *SIG1* gene results in decreased transcript accumulation of mostly PSI (*psa*) genes, which is accompanied by a reduction in the amount of PSI (Tozawa *et al.*, 2007). Transcript accumulation of *psbB* and *psbE* operons, encoding PSII reaction center core polypeptides, also decreases in the rice *sig1* knockout, but to a lesser degree compared with the *psa* genes. A similarly altered chloroplast transcription is seen in a *SIG1* knockout mutant of the liverwort *Marchantia* (Ueda *et al.*, 2013). ChIP of Arabidopsis SIG-bound promoter regions shows enrichment of multiple chloroplast operons including genes for both photosystems and the Rubisco large subunit (*RbcL*) (Hanaoka *et al.*, 2012). Arabidopsis SIG1 has further been shown to become phosphorylated with important regulatory implications for PSI gene transcription (Shimizu *et al.*, 2010). To further understand the role of SIG1 in basal and regulated transcription of chloroplast genes, we analyzed two independent Arabidopsis *SIG1* T-DNA insertion mutants.

## Materials and methods

### Plant growth conditions

*Arabidopsis thaliana* (Col-0) wild-type and mutant plants were grown from seeds on soil at 23 °C under a photon flux density of 150 µmol m^–2^ s^–1^ with an 8 h light and 16 h dark photoperiod, unless otherwise specified. For the light switch time-course experiment, wild-type Arabidopsis, *sig1-1*, *sig1-2*, and *phyB* null mutant (*phyB-9*; harboring a premature stop codon) seedlings were grown in white light (150 µmol m^–2^ s^–1^; 16 h light) for 7 d and then transferred to light 1 (L1) or light 2 (L2) cabinets and allowed to acclimate for 4 d. At the end of the fourth day, the lights were switched (i.e. from L1 to L2, or vice versa). Leaf samples were collected before the light switch (zero time) and at 4, 8, 24, 28, and 32 h after the light switch.

### Light 1 and 2

L1 was provided with Narva 18 W/015 red fluorescent strip lamps wrapped with a layer of plasa red filter (LEE 029). White fluorescent lamps (Philips Master TL-D 18 W/827) covered with a layer of burnt yellow filter (LEE 770) produced L2. Photon flux density at the leaf level was ~6 µmol m^–2^ s^–1^ in L1 and ~12 µmol m^–2^ s^–1^ in L2. L1 and L2 were made available on a photoperiod of 16 h light and 8 h dark. The specific action of L1 and L2 on PSI and PSII, respectively, were confirmed with measurement of the room temperature variable fluorescence yield associated with state transitions (data not shown).

### Genotyping of SALK T-DNA lines

The SALK lines used in this study were genotyped for homozygous T-DNA insertion by using genomic and T-DNA cassette primers. Genomic DNA were isolated from plant leaf tissues by a protocol adapted from the Dellaporta method (Dellaporta *et al.*, 1983). A reverse transcription–PCR (RT–PCR) was further used to check SIG1 transcript accumulation in the T-DNA lines. Amplification of the housekeeping gene *Actin8* served as a control for RNA integrity. Total RNA was isolated from leaves by using the TRIzol reagent (Invitrogen) as per the manufacturer’s instructions. The cDNA was synthesized from 1 µg of total RNA with the RevertAid First Strand cDNA synthesis kit (Fisher Scientific) using an oligo(dT)_18_ primer. *SIG1* and *Actin8* transcripts were further detected by a Taq PCR using gene-specific primers. Sequences of all primers used are provided in Supplementary Table S1 at *JXB* online.

### SIG1 protein quantification

The level of SIG1 protein was analyzed by a polyclonal antibody raised against the Arabidopsis SIG1. Total leaf protein was extracted from the wild type and *sig1* mutants, and the protein concentration was determined with the Pierce BCA kit (Thermo Scientific). Equal amounts of protein samples were subjected to 11.5% (w/v) SDS–6 M urea–PAGE and electroblotted onto a polyvinylidene fluoride (PVDF) membrane (Immobilon-P, Millipore). The membrane was then blocked with 5% (w/v) non-fat dry milk (Bio-Rad) overnight at 4 °C, washed, and probed with the SIG1 primary antibody for 90 min at room temperature. Horseradish peroxidase-conjugated anti-rabbit secondary antibody (NA934, GE Healthcare) was used in the immunodetection of SIG1. Immunoreactive bands were visualized on a ChemiDoc MP imager (Bio-Rad) using a chemiluminescence detection reagent (Clarity Western ECL Substrate, Bio-Rad). A monoclonal plant actin antibody (A0480, Sigma) was used as a loading control and was detected using an anti-mouse secondary antibody (NA931, GE Healthcare). Band intensities of SIG1 and actin were analyzed by the ImageJ software.

### Quantitative real-time PCR (qRT-PCR)

Total RNA was isolated from the leaves of 11- to 14-day-old light switch samples by using the TRIzol reagent. RNA was treated with RNase-free DNase (New England Biolabs) to eliminate possible DNA contamination. qRT-PCR was performed with a one-step QuantiTech SRBR Green RT-PCR kit from Qiagen in a StepOnePlus thermocycler (Applied Biosystems). The amplification efficiency of each primer pair (Supplementary Table S1) was checked by a 64-fold serial dilution of the template, and the *R*^2^ value of each primer pair was found to be ≥0.99. The expression values of target genes were normalized to both total RNA and endogenous *Actin8* control. The relative changes in gene expression were analyzed by a 2^–ΔΔCt^ method.

For qRT-PCR of DCMU [3-(3,4-dichlorophenyl)-1,1-dimethylurea]-treated samples, excised leaves from 5- to 6-week-old wild-type plants were vacuum infiltrated with 10 µM DCMU and incubated under 150 µmol m^–2^ s^–1^ white light for 6 h while being floated in an isotonic buffer containing 0.4 M sorbitol, 20 mM tricine (pH 8.4), 10 mM EDTA, 10 mM NaHCO_3_, 0.15% (w/v) BSA, and 10 µM DCMU. At the end of the DCMU treatment, total RNA was isolated and qRT-PCR was done as before.

### SIG1 complementation

*SIG1* was complemented in the SALK_147985c line using *Agrobacterium*-mediated gene transfer. The coding sequence of *SIG1* was cloned into a customized pCC2134_BAR expression vector (see Supplementary Table S1 for the primers used) and the resulting gene construct was delivered to Arabidopsis plants by the floral dip method. Transformants were subsequently screened by the Basta (bar) selection marker. qRT-PCR of selected nuclear and chloroplast genes was performed in complemented lines as before.

## Results

### Characterization of the *SIG1* T-DNA insertion mutants

**Fig. 1.**
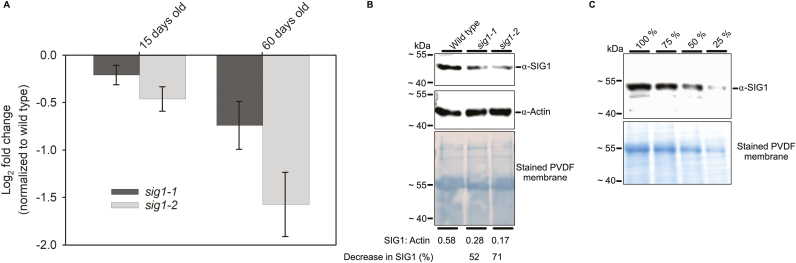
T-DNA insertional mutagenesis decreases *SIG1* transcript and protein levels. (A) *SIG1* transcript abundance in *sig1-1* and *sig1-2* mutants as quantified by qRT-PCR. The log2 fold change after normalization with the wild type is shown. Error bars represent ±SE of the mean of four biological replicates. (B) SIG1 protein level as estimated by immunoblotting. Representative SIG1 and actin blots are shown with the corresponding stained PVDF membrane. Both SIG1 and actin are detected on the same membrane. Numbers below each lane denote the ratio of SIG1 to actin band intensity. The percentage decreases in SIG1 relative to the wild-type control are also given. The full uncropped versions of SIG1 and actin immunoblots are given in Supplementary Fig. S5. (C) An immunoblot of SIG1 with serial dilutions of the wild-type sample. The corresponding stained membrane is also shown. Molecular weight markers are indicated on the left. The mature SIG1 protein has a predicted mol. wt of 54 kDa. The SIG1 protein, however, runs on an 11.5% (w/v) SDS–6 M urea–PAGE gel with an apparent mol. wt of ~49 kDa.

Two Arabidopsis T-DNA lines harboring insertions in the *SIG1* gene locus *At1g64860* were obtained from the ABRC. These mutant lines, SALK_147985c and CS371990, are hereafter simply referred to as *sig1-1* and *sig1-2*, respectively. Figure 1A shows *SIG1* transcript abundance in these mutants as quantified by qRT-PCR. Both mutants show decreased accumulation of the *SIG1* transcript. The *SIG1* transcript reduction is, however, more marked in *sig1-2*. *sig1-1* is a confirmed homozygous SALK line with a T-DNA insertion in the 3'-untranslated region (UTR) of the *SIG1* gene, while *sig1-2* is a confirmed homozygous GABI-Kat line that carries an insertion within the penultimate exon (Supplementary Figs S1, S2). The different extent of *SIG1* transcript reduction in the two mutants (Fig. 1A) is consistent with their unique T-DNA insertion sites. A T-DNA within the coding sequence is expected to disrupt transcript accumulation more severely, as seen in *sig1-2*, than an insertion within the 3'-UTR as in *sig1-1*. The varying degrees of transcript reduction further manifest at the SIG1 protein level in both mutants, as quantified by immunoblotting with an anti-SIG1 antibody (Fig. 1B). Though the SIG1 protein level decreases more noticeably in *sig1-2*, it is interesting to note that both mutants, regardless of T-DNA insertion, are able to accumulate apparently full-length SIG1 proteins. For *sig1-1*, this observation is consistent with its 3'-UTR location of T-DNA. For *sig1-2*, the T-DNA insertion is predicted to shorten the SIG1 protein by 62 amino acids (~6 kDa). A genotyping RT–PCR, designed to amplify the coding sequence of the mature SIG1 protein, however, shows that this does not happen (Supplementary Fig. S1D). The *sig1-2* mutant indeed accumulates a faint amplificate of the right size, indicating that it is somehow able to splice out the T-DNA from the precursor mRNA and produce the full-length SIG1 protein—albeit less efficiently (Fig. 1; Supplementary Fig. S1D). *sig1-1* and *sig1-2* thus appear not to be true knockout mutants of *SIG1* but rather knockdown lines.

### Chloroplast gene targets of SIG1

**Fig. 2.**
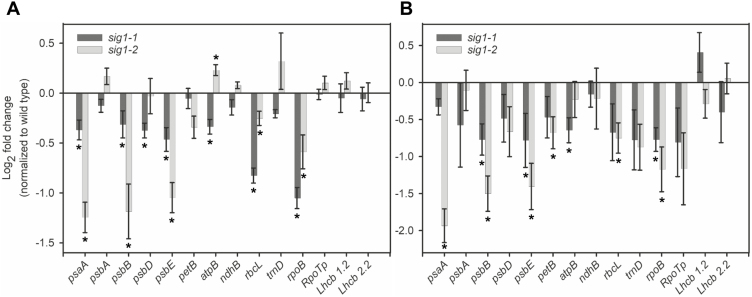
Abundance of selected chloroplast and nuclear gene transcripts in *sig1* mutants. Transcript abundance in 15- (A) and 60-day-old plants (B). The log2 fold change after normalization with the wild type is shown. The asterisks indicate statistically significant changes (*P*-value <0.05 on a one- or two-tailed distribution). Error bars represent ±SE of the mean of four biological replicates.

Since expression of chloroplast genes may correlate with the expression of their corresponding sigma factors, we analyzed transcript accumulation of all six sigma factor genes in leaves taken from different developmental stages of Arabidopsis. Plants were grown in short-day conditions to promote vegetative growth and to delay flowering. *SIG1* seems to be maximally expressed in 60-day-old plants (Supplementary Fig. 3). Other sigma factors also show unique development-specific expression profiles, the implications of which for SIG1 function and chloroplast transcription in general are discussed further on. In our analysis of *SIG1* gene targets, we have thus included a 60-day-old sample in addition to the 15-day-old young plants. Transcript abundance of all major chloroplast gene operons were quantified in wild-type, *sig1-1*, and *sig1-2* plants by qRT-PCR. In 15-day-old *sig1-1*, transcript levels of the following chloroplast operons were significantly decreased compared with the wild type (*P*-value <0.05): *psaA*, *psbD*, *psbE*, *rbcL*, and *rpoB* (Fig. 2A). If we reanalyze the gene expression data using a one-tailed *t*-test distribution with the expectation that the lack of SIG1 affects chloroplast genes only in one direction (i.e. lower than the wild type), then the *psbB* and *atpB* operons also join the list of down-regulated genes (*P*-value <0.05). The *rpoB* gene, encoding the β-subunit of PEP, has been shown to be transcribed predominantly by the major chloroplast NEP isoform RpoTp in multiple plant species (Silhavy and Maliga, 1998; Zhelyazkova *et al.*, 2012). We therefore examined whether the observed reduction in *rpoB* transcript arises from a corresponding decrease in *RpoTp* transcript. This, however, seems not to be the case as the *RpoTp* transcript level remains unaffected in 15-day-old *sig1-1* (Fig. 2).

The activities of chloroplast sigma factors SIG2 and SIG6 have been shown to generate distinct plastid to nuclear retrograde signals for the regulation of photosynthesis-associated nuclear genes (PhANGs) (Woodson *et al.*, 2013). To examine whether the SIG1 activity is the source of any retrograde signal, the expression of two canonical PhANG genes *Lhcb 1.2* and *Lhcb 2.2* was checked in *sig1* mutants. The transcript levels of these two genes were unchanged in 15-day-old *sig1-1* (Fig. 2A). The 15-day-old *sig1-2* mutant shows a similar set of chloroplast transcripts, with the levels of *psaA*, *psbB*, *psbE*, and *rpoB* significantly decreased, more so than in the *sig1-1* mutant (Fig. 2A). A one-tailed *t*-test (*P*-value <0.05) would add *rbcL* to the list of down-regulated genes in *sig1-2*, but the *psbD* and *atpB* transcripts seem to decrease only in *sig1-1*. None of the nuclear gene transcripts shows any change in *sig1-2*, as with *sig1-1* (Fig. 2A). The 60-day-old *sig1-1* shows decreased accumulation of *psbB*, *psbE*, *atpB*, and *rpoB* transcripts on a two- or one-tailed *t*-test distribution (*P*-value <0.05), while the similarly aged *sig1-2* mutant reveals reduction in *psaA*, *psbB*, *psbE*, *petB*, *rbcL*, and *rpoB* transcripts (*P*-value <0.05; two-tailed *t*-test) (Fig. 2B). As with 15-day-old plants, neither of the 60-day-old mutant plants shows any change in nuclear gene transcripts (Fig. 2B).

**Fig. 3.**
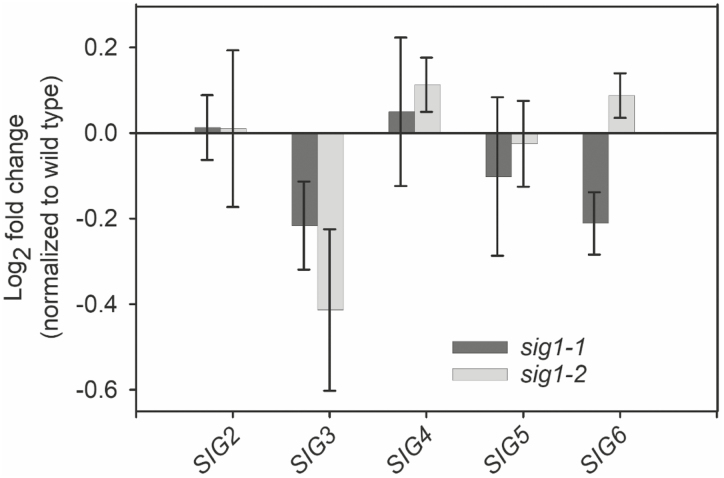
Unchanged *SIG2*–*SIG6* expression in 15-day-old *sig1* mutants. The *sig1* mutation does not result in any statistically significant changes in the transcript abundance of other sigma factors. The log2 fold change after normalization with the wild type is shown. Error bars represent ±SE of the mean of four biological replicates.

Overall, the magnitude of the reduction in chloroplast gene transcripts parallels the SIG1 protein level in each mutant (Fig. 1), with less SIG1 protein leading to lower accumulation of chloroplast transcripts in both 15- and 60-day-old mutants (Fig. 2). To check whether the decreased accumulation of SIG1 protein triggers any pleiotropic or compensatory response on the gene expression of other sigma factors, we analyzed their transcript abundance in 15-day-old *sig1-1* and *sig1-2* mutants (Fig. 3). Compared with the wild type, neither of the mutants shows any statistically significant change in transcript accumulation of *SIG2*, *SIG3*, *SIG4*, *SIG5*, and *SIG6* (Fig. 3).

### Light quality acclimation involves transcriptional control of the nuclear *SIG1* gene

**Fig. 4.**
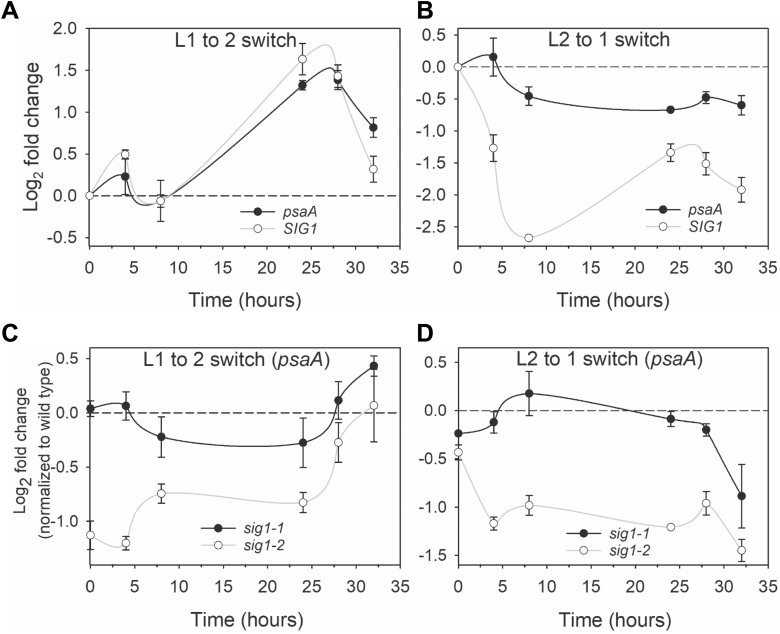
Transcript accumulation kinetics of *psaA* and *SIG1* genes. (A and B) Transcript accumulation kinetics of *psaA* and *SIG1* in the wild type as quantified with qRT-PCR. Experimental conditions are switch from light 1 (L1) to light 2 (L2), and vice versa. The log2 fold change in transcript abundance is plotted against time. The time point at which lights are switched is taken as zero time and the concomitant up- or down-regulation is calculated by taking the expression at the time of light switch (zero time) as baseline. (C and D) Transcript accumulation kinetics of *psaA* in *sig1* mutants. Each data point represents log2 fold change relative to the wild-type control. Error bars represent ±SE of the mean of three biological replicates.

L1, enriched in far red, preferentially excites PSI and thereby oxidizes the plastoquinone (PQ) pool. An oxidized PQ pool leads to phosphorylation of SIG1 and the phospho-SIG1 represses *psaA* gene transcription (Shimizu *et al.*, 2010). L2 is a short wavelength light that selectively excites PSII and thus reduces the PQ pool. A reduced PQ pool releases the repression on *psaA* gene transcription by dephosphorylating and/or degrading phosphorylated SIG1 (Shimizu *et al.*, 2010). To further understand the involvement of SIG1 in the transcriptional control of *psaA*, we measured the kinetics of *psaA* and *SIG1* transcript accumulation in the wild type in response to changes in light quality. Figure 4A shows *psaA* transcript accumulation kinetics of the wild type in response to an L1 to L2 switch. As expected, the *psaA* transcript level increases in PSII-specific L2. Interestingly, the *SIG1* gene shows similar transcript accumulation kinetics to *psaA* (Fig. 4A). In the opposite light switch, namely when the PSII-specific L2 is replaced with PSI-specific L1, the amount of *psaA* transcript decreases as the light begins to favor PSI (Fig. 4B). The *SIG1* transcript shows a similar reduction in L1 but the magnitude of its decrease is much higher than that of *psaA* (Fig. 4B).

To shed further light on the significance of concerted *psaA* and *SIG1* transcript accumulation, we monitored *psaA* transcript accumulation kinetics in *sig1* knockdown mutants. Figure 4C shows *psaA* transcript accumulation in response to an L1 to L2 switch. Compared with the wild type, *sig1-2* has significantly less *psaA* transcript at the end of L1 illumination (time zero). After switch to L2, the *psaA* transcript level begins to increase but it takes nearly 32 h for the mutant to reach the wild-type level. The slower *psaA* transcriptional response of *sig1-2* is consistent with its substantially lower SIG1 protein amount and its inability to further increase the SIG1 protein level promptly in L2. Interestingly, *psaA* transcript accumulation kinetics of the *sig1-1* mutant were similar to those of the wild type except at 8 h and 24 h after the light switch. This nearly wild-type-like response of *sig1-1* is compatible with its weaker *SIG1* T-DNA allele. In the reciprocal light shift (i.e. L2 to L1 switch), the *psaA* transcript decreases more than that of the wild type in both mutants at 32 h after the light switch (Fig. 4D). However, the level of *psaA* transcript before the light switch (i.e. at time zero in L2) is also lower than that of the wild type in both mutants (Fig. 4D).

### Redox- and light quality-dependent regulation of *SIG1* gene transcription

To identify the factors that allow coordinated expression of the nuclear *SIG1* gene with its chloroplast *psaA* target gene, we first checked the role of the PQ pool redox state. This is based on two considerations: (i) the *psaA* transcriptional response is governed by the PQ pool redox state (Pfannschmidt *et al.*, 1999); and (ii) the PQ pool redox signal affects transcription of multiple nuclear genes (Fey *et al.*, 2005). To test the role of the PQ pool, we first chemically altered its redox state with the electron transport inhibitor DCMU. DCMU binds to the Q_B_-site of PSII and thus prevents reduction of PQ by PSII. The PQ pool therefore exists in a more oxidized state in DCMU-treated plants. Leaves from mature wild-type plants were infiltrated with DCMU and illuminated. The *SIG1* transcript level in DCMU-treated samples decreased by 0.34±0.06-fold on a log2 scale (Supplementary Fig. S4), which is equivalent to a reduction of 21.2±4.0% relative to the untreated sample. Both DCMU and L1 (Fig. 4B; Supplementary Fig. S4) thus appear to decrease *SIG1* transcript abundance. The effect of DCMU, however, seems modest compared with the large drop in *SIG1* transcript abundance in L1 (Fig. 4B; Supplementary Fig. S4), raising the possibility that other factors besides the PQ pool redox state may also regulate the nuclear *SIG1* gene expression.

**Fig. 5.**
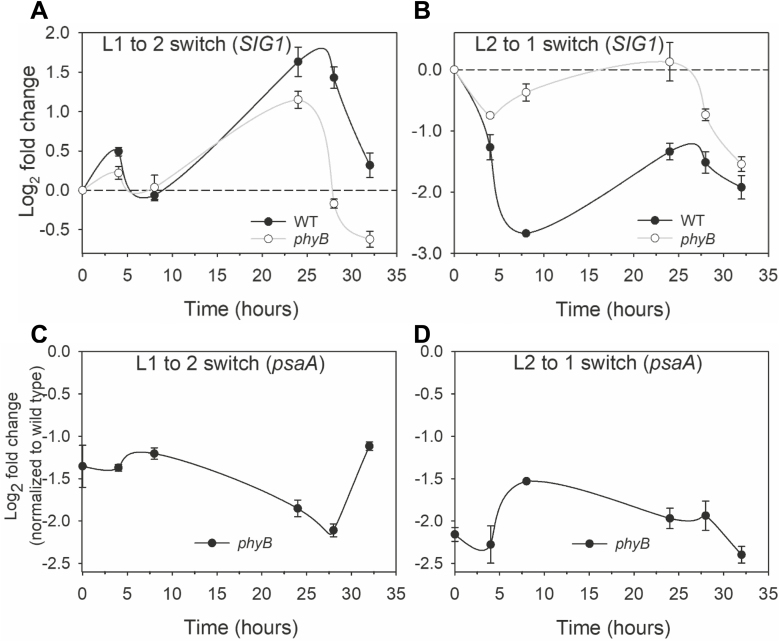
Light quality regulation of nuclear *SIG1* and chloroplast *psaA* genes. (A and B) *SIG1* transcript accumulation kinetics in the wild type and *phyB* mutant. The wild-type data are replotted from Fig. 4. (C and D) The log2 fold change in transcript accumulation of *psaA* in the *phyB* mutant is shown relative to the wild-type control. Error bars represent ±SE of three biological replicates.

Since phytochromes mediate many plant responses to light, we further checked *SIG1* transcript accumulation kinetics in a phytochrome mutant (*phyB*). Figure 5A shows the response of the *phyB* mutant to the L1 to L2 switch. The level of *SIG1* transcript in *phyB* fails to reach wild-type levels at 24, 28, and 32 h after the light switch. The aberrant *SIG1* transcriptional response of *phyB* is more noticeable in the opposite L2 to L1 switch, where the initial rapid fall component of the response is nearly absent in the mutant (Fig. 5B). Significant differences from the wild type are also apparent at other time points (Fig. 5B). To determine how this impaired *SIG1* transcriptional response affects *psaA* transcript accumulation kinetics in *phyB*, we quantified its *psaA* transcript level (Fig. 5C, D). The *psaA* transcript level in *phyB* is significantly lower than in the wild type in both L1 to L2 and L2 to L1 conditions (Fig. 5C, D). It is notable that the *psaA* transcript hardly changes after 32 h in L2 or L1 from the level at zero time (before the light switch) (Fig. 5C, D). This stands in contrast to *sig1* knockdown mutants, which are able to catch up with the wild type eventually (Fig. 4C, D).

## Discussion

### SIG1 initiates transcription of a specific subset of chloroplast genes

With a eubacterial RNA polymerase, six sigma factors, and two phage-type RNA polymerases, the transcriptional machinery of chloroplasts seems an overelaboration for just a handful of genes. It nevertheless appears that this elaborate cellular machinery forms the basis of a complex transcriptional program in chloroplasts that is both developmentally attuned and environmentally responsive. This transcriptional robustness seems to be built on an intricate division of labor among chloroplast sigma factors. SIG2 is required for transcription initiation of *psbA*, *psaJ*, and several tRNAs, including glutamyl-tRNA (*trnE*). *trnE* is a precursor for the biosynthesis of tetrapyrroles—chlorophylls and hemes—and therefore *SIG2* knockout mutants show a pale phenotype (Hanaoka *et al.*, 2003; Nagashima *et al.*, 2004). Because of its involvement in *trnE* transcription and, as a result, tetrapyrrole metabolism, SIG2 activity seems to be the source of a chloroplast to nuclear retrograde signal (Woodson *et al.*, 2013).

Available data indicate that SIG3 initiates transcription of only *psbN* and *atp* (ATPase) genes (Zghidi *et al.*, 2007; Malik Ghulam *et al.*, 2012), and SIG4, likewise, has a marginal role, transcribing only the *ndhF* gene encoding the F subunit of the NDH complex (Favory *et al.*, 2005). SIG5 has a 2-fold function in chloroplast transcription. Binding to a blue light-responsive promoter, it transcribes the *psbD* gene that encodes the D2 protein of PSII (Tsunoyama *et al.*, 2004). SIG5 further functions as a nuclear-encoded timing signal that generates a circadian rhythm in chloroplast gene transcription (Noordally *et al.*, 2013). The null mutant of *SIG6* exhibits a cotyledon-specific pale green phenotype with delayed chloroplast development (Ishizaki *et al.*, 2005). Transcripts of several photosynthetic genes are decreased in the *sig6* mutant. The SIG6 activity, like that of SIG2, seems to generate a nuclear gene regulatory retrograde signal (Woodson *et al.*, 2013). The age-dependent expression profiles of nuclear sigma factors (Supplementary Fig. S3) broadly reflect their division of labor in plastid transcription. Out of the six factors, SIG3 shows the lowest expression in all stages of development, probably consistent with its limited functional role. The other sigma factors exhibit relatively high expression, peaking especially in mature plants, in line with their major transcriptional roles in chloroplasts. An exception is SIG4, which seems to act only at the *ndhF* gene promoter and yet displays high expression in all growth stages. It is possible that SIG4 has a wider functional role than currently realized with as yet unknown target genes.

The current understanding of the role of Arabidopsis SIG1 is largely based on a ChIP sequencing (ChIP-Seq) study and on inference from the rice *sig1* knockout mutant (Tozawa *et al.*, 2007; Hanaoka *et al.*, 2012). The *sig1-1* and *sig1-2* mutants described in the current study have been used before with the assumption that they are complete knockout mutants (Lai *et al.*, 2011; Woodson *et al.*, 2013). As we show here, these mutants are rather knockdown lines of *SIG1* (Fig. 1). The *sig1-2* mutant, which contains the T-DNA insert within the coding sequence, appears to be a stronger knockdown allele of *SIG1* than the *sig1-1* mutant that harbors the insertion in the 3'-UTR (Fig. 1). The transcript abundance of chloroplast genes in *sig1-1* and *sig1-2* suggests that SIG1 is one of the sigma factors that initiate transcription of *psaA*, *psbB*, *psbE*, *rbcL*, and *rpoB* operons in Arabidopsis (Fig. 2). The *psbD* and *petB* transcripts also show reduction, but only in one out of the four samples analyzed (Fig. 2). Likewise, the *atpB* transcript seems to decrease only in *sig1-1* (Fig. 2). The *sig1-1* mutant shows a slight variation between 15- and 60-day-old samples in the set of significantly affected chloroplast genes, consistent with its weaker T-DNA allele. Interestingly, our list of *SIG1* gene targets overlaps significantly with the operons identified in the ChIP-Seq study, which shows enrichment of *SIG1* in the gene promoters of *psaA*, *psbB*, *psbE*, *rbcL*, and *clpP* (Hanaoka *et al.*, 2012). The *ClpP* gene, which encodes the chloroplast ClpP protease, was not analyzed in our study. Our gene expression data go beyond the ChIP-Seq study by identifying the *rpoB* gene as a likely target of SIG1 in Arabidopsis (Fig. 2).

The plant *rpoB* gene is mostly transcribed by the NEP isoform RpoTp (Hricova, 2006). Given the unchanged *RpoTp* gene expression in mutants (Fig. 2), it is unclear how the deficiency in SIG1, a PEP subunit, would cause this phenotype. Two scenarios might explain this odd observation. The first is an intriguing possibility that RpoTp utilizes SIG1 for initiation of *rpoB* transcription. It has been suggested that the plant phage-type polymerases, similar to yeast and human mitochondrial phage-type polymerases, require additional factors to melt gene promoters. None has, however, been identified in plants thus far (Börner *et al.*, 2015). Interestingly, multiple sigma factors including SIG1 and SIG5 seem to be targeted to plant mitochondria and are co-purified with the mitochondrial phage-type polymerase (Beardslee *et al.*, 2002). It is thus possible that SIG1 functions as an accessory subunit of RpoTp for the transcription initiation of *rpoB* in plant chloroplasts. A second explanation for the decreased *rpoB* transcription is that the *rpoB* operon contains an as yet uncharacterized SIG1-dependent PEP promoter. Such a PEP promoter may create a positive feedback loop in PEP β-subunit expression, allowing rapid developmental and environmental acclimation of chloroplast gene transcription.

Since *rpoB* encodes a core subunit of PEP, it should be considered whether a decrease in its transcription and in turn the PEP content explains the observed reduction of chloroplast transcripts in *sig1* mutants (Fig. 2). The unchanged expression of many PEP-dependent operons in *sig1-1* and *sig1-2*, however, rules out this possibility (Fig. 2), as a deficiency in PEP would affect the overall chloroplast transcription rather than that of a specific subset of chloroplast genes. In fact, a general decrease in chloroplast transcription, including that of the exclusively PEP-transcribed *psbA* and *rbcL* genes, has been reported in an Arabidopsis *RpoTp* null mutant. This was attributed to a diminished PEP level resulting from the decreased transcription of *rpoB* (Courtois *et al.*, 2007). The *RpoTp* knockout mutant that produces very little *rpoB* transcript and our *sig1* knockdown mutants, which show only a 2-fold reduction in *rpoB*, thus seem to possess two distinct chloroplast transcriptional phenotypes.

**Fig. 6.**
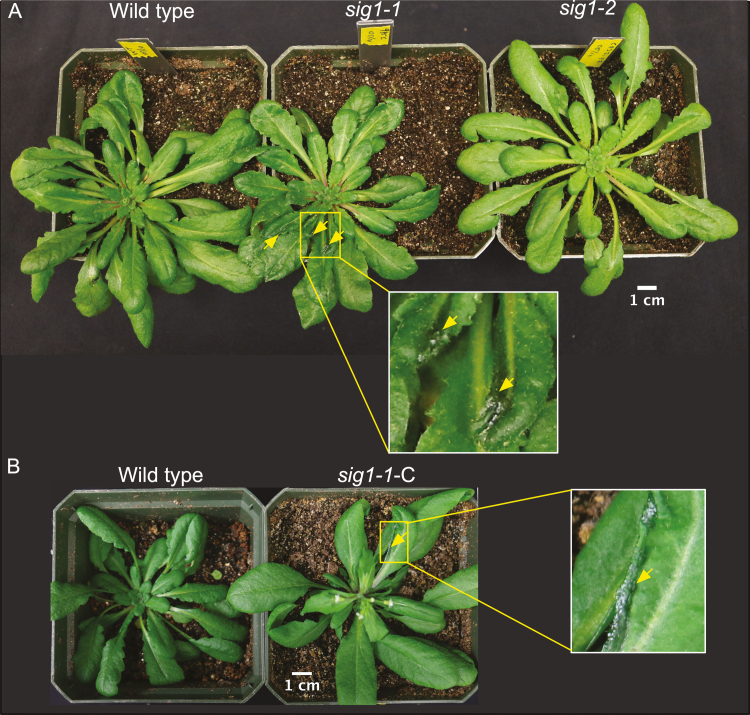
The *sig1-1* mutant shows a curly leaf phenotype that is unrelated to SIG1 deficiency. (A) RGB images of mature wild-type, *sig1-1*, and *sig1-2* plants. The curly, deformed leaf areas in s*ig1-1* are indicated by yellow arrowheads. The inset shows an enlarged view of the lesion. (B) Images of the wild type and complemented *sig1-1* mutant (*sig1-1-C*). Scale bars are shown.

**Fig. 7.**
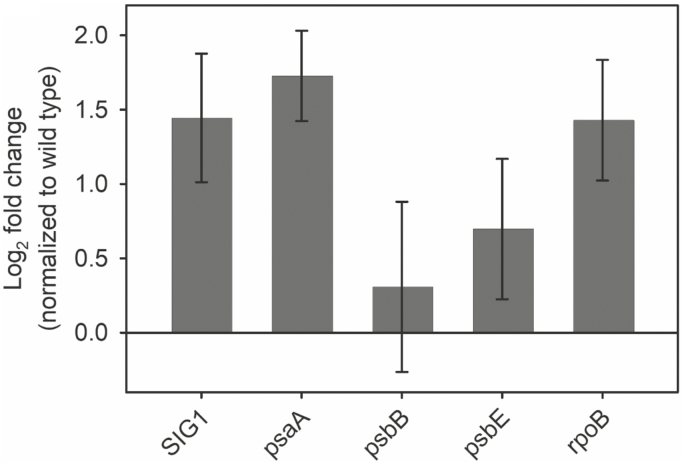
Abundance of selected gene transcripts in 60-day-old *sig1-1-C*. The log2 fold change relative to the wild type is shown. Error bars represent ±SE of four biological replicates.

Curiously, the mature *sig1-1* plants show a unique leaf phenotype in which some leaves become curly and deformed (Fig. 6). This phenotype, however, must be due to a second T-DNA insertion in a separate gene locus since the *sig1-2* mutant does not show any such abnormal leaves (Fig. 6A). To further test whether the ‘curly’ leaf phenotype has anything to do with SIG1, we complemented the *SIG1* gene in *sig1-1* on a *Cauliflower mosaic virus* (CaMV) 35S promoter. The ‘curly’ leaf phenotype, however, could not be complemented (Fig. 6B), supporting the distinct genetic nature of this anomaly. *SIG1* overexpression instead seems to complement the chloroplast transcriptional phenotype of *sig1-1*, with some gene targets of SIG1 showing increased transcript accumulation compared with the wild type (Fig. 7).

### SIG1 in photosynthetic control of PSI gene transcription

Besides their intrinsic preference for certain gene promoters, a property common to all sigma factors, chloroplast sigma factors further possess a unique regulatory feature in the form of phosphorylation. Protein phosphorylation has been shown to modulate the activity of SIG1 and SIG6 (Schweer *et al.*, 2010*b*; Shimizu *et al.*, 2010). SIG6 is phosphorylated by a chloroplast casein kinase known as the plastid transcription kinase (PTK). Phosphorylated SIG6 initiates transcription of the *atpBE* operon from its PEP promoter. The functional context of this regulation, however, remains unclear. Phosphorylation of SIG1, on the other hand, is associated with the transcriptional control of the PSI reaction center operon *psaAB* during a remarkable light quality acclimatory response known as photosystem stoichiometry adjustment. Changes in the wavelength of light that selectively excites PSII and PSI, and therefore the redox state of the PQ pool located between the two photosystems, initiates stoichiometric changes in the two photosystems so as to correct any imbalance in light energy conversion at either photosystem. It has been recognized that the adjustment of photosystem stoichiometry occurs mostly through changes in PSI amount and that the transcriptional regulation of *psaAB* is critical to this regulation (Murakami *et al*., 1997*a*, *b*; Pfannschmidt *et al.*, 1999). The phosphorylation of SIG1 seems to provide a mechanism to connect the PQ pool redox state with transcription of the *psaAB* gene (Shimizu *et al.*, 2010). The oxidized PQ pool, as under PSI-specific far-red light, leads to the phosphorylation of SIG1, which then represses *psaAB* transcription initiation and, as a result, lowers the amount of PSI. In the opposite light condition (i.e. under PSII-specific short wavelength illumination), the PQ pool becomes more reduced. Under this condition, phospho-SIG is thought to be either dephosphorylated or degraded. This releases the repression on *psaAB* transcription, increasing the amount of PSI. A key observation that links SIG1 phosphorylation with photosystem stoichiometry adjustment is the demonstration that the phosphorylation site mutant of *SIG1* is unable to repress *psaAB* in PSI light.

An Arabidopsis knockout mutant of the chloroplast sensor kinase (CSK), a modified bacterial-type two-component sensor kinase, is similarly unable to repress *psaAB* in PSI light (Puthiyaveetil *et al.*, 2008). CSK has therefore been suggested as the SIG1 kinase (Puthiyaveetil *et al.*, 2013). Many key aspects of the CSK–SIG1 signaling pathway, however, remain unclear. For example, in *csk* knockout mutants, the amount of *psaAB* transcript reaches the wild-type level after nearly 24 h of misregulation (Puthiyaveetil *et al.*, 2008). This suggests that SIG1 phosphorylation by CSK could be just one facet of photosystem stoichiometry adjustment and that there are other as yet unknown layers of regulation in this important photosynthetic light acclimatory response. Towards this, we identify that the *psaAB* transcriptional control requires a complementary regulation of nuclear *SIG1* gene transcription (Fig. 4). It thus appears that *psaAB* gene regulation during photosystem stoichiometry adjustment requires not only post-translational control of SIG1 activity through phosphorylation but also regulation of the amount of SIG1 protein via changes in *SIG1* gene transcription. The apparent emphasis on regulation of the SIG1 protein level further raises the question of whether the phosphorylation is indeed a tag for degradation of SIG1. If correct, this would suggest that both phosphorylation and *SIG1* transcriptional control act in concert to decrease the SIG1 protein level and, as a result, *psaAB* transcription in PSI light (Fig. 4).

The use of the electron transport inhibitor DCMU shows that the redox state of the PQ pool, the very signal that initiates *psaAB* transcriptional changes, also controls nuclear *SIG1* gene transcription. The PQ redox state is a well-known retrograde signal affecting nuclear photosynthetic genes and it is not surprising that it may also regulate the *SIG1* gene (Pfannschmidt *et al.*, 2001). The true extent of the PQ control of *SIG1* transcription remains to be seen as we find only a modest effect in our experimental conditions (Supplementary Fig. S4). The *phyB* mutant nevertheless shows that light quality *per se* is the most effective signal driving *SIG1* transcription, with the *phyB* mutant lacking some crucial aspects of *SIG1* and *psaA* gene regulation (Fig. 5). The dual redox and light control is not unique to SIG1, as the transcription of *SIG5* is regulated in part by redox- and phytochrome-mediated light signaling (Mellenthin *et al.*, 2014). Some of the complex kinetics in *psaA* and *SIG1* transcript accumulation are probably the result of both redox and light signals exerting their influence simultaneously (Figs 4, 5). These redox and light effects seem further superimposed on an endogenous rhythm in transcription as driven by nuclear and chloroplast circadian timekeepers (Figs 4, 5). Finally, it is pertinent to ask what purpose these seemingly redundant pathways of chloroplast *psaAB* gene regulation serve. It is likely that the redox-controlled and CSK-mediated SIG1 phosphorylation is a fast-acting regulatory mechanism, while the regulation through nuclear *SIG1* transcriptional control is a long-term strategy. The mostly light quality-dependent regulation of *SIG1* gene transcription (Fig. 5) may further provide a crucial, fail-safe input into the predominantly redox-controlled *psaAB* gene transcription.

## Supplementary data

Supplementary data are available at *JXB* online.

**Fig. S1.** Genotyping of *sig1-1* and *sig1-2* mutants.

**Fig. S2.** T-DNA insertion sites in *sig1-1* and *sig1-2* mutants.

**Fig. S3.** Developmental profile of sigma factor gene expression.

**Fig. S4.** The effect of DCMU on *SIG1* gene expression.

**Fig. S5.** Full uncropped western blots of SIG1 and actin.

**Table S1.** Primers used in this study.

erz464_suppl_Supplementary_Data

erz464_suppl_Supplementary_Figure_S1-S5

erz464_suppl_Supplementary_Table_S1
