# Supplementary material for: Sigma factor 1 in chloroplast gene transcription and photosynthetic light acclimation
Source: J Exp Bot. 2019 Oct 23;71(3):1029–38. doi: 10.1093/jxb/erz464 (PMC6977190; doi:10.1093/jxb/erz464)
Supplement: erz464_suppl_Supplementary_Data [file erz464_suppl_supplementary_data.docx]

**Supplementary data**

**Fig. S1.** Genotyping of *sig1-1* and *sig1-2* mutants

**Fig. S2.** T-DNA insertion sites in *sig1-1* and *sig1-2* mutants

**Fig. S3.** Developmental profile of sigma factor gene expression

**Fig. S4.** The effect of DCMU on *SIG1* gene expression

**Fig. S5.** Full uncropped western blots of SIG1 and Actin

**Table S1.** Primers used in this study
