# Supplementary material for: Sigma factor 1 in chloroplast gene transcription and photosynthetic light acclimation
Source: J Exp Bot. 2019 Oct 23;71(3):1029–38. doi: 10.1093/jxb/erz464 (PMC6977190; doi:10.1093/jxb/erz464)
Supplement: erz464_suppl_Supplementary_Figure_S1-S5 [file erz464_suppl_supplementary_figure_s1-s5.pdf]

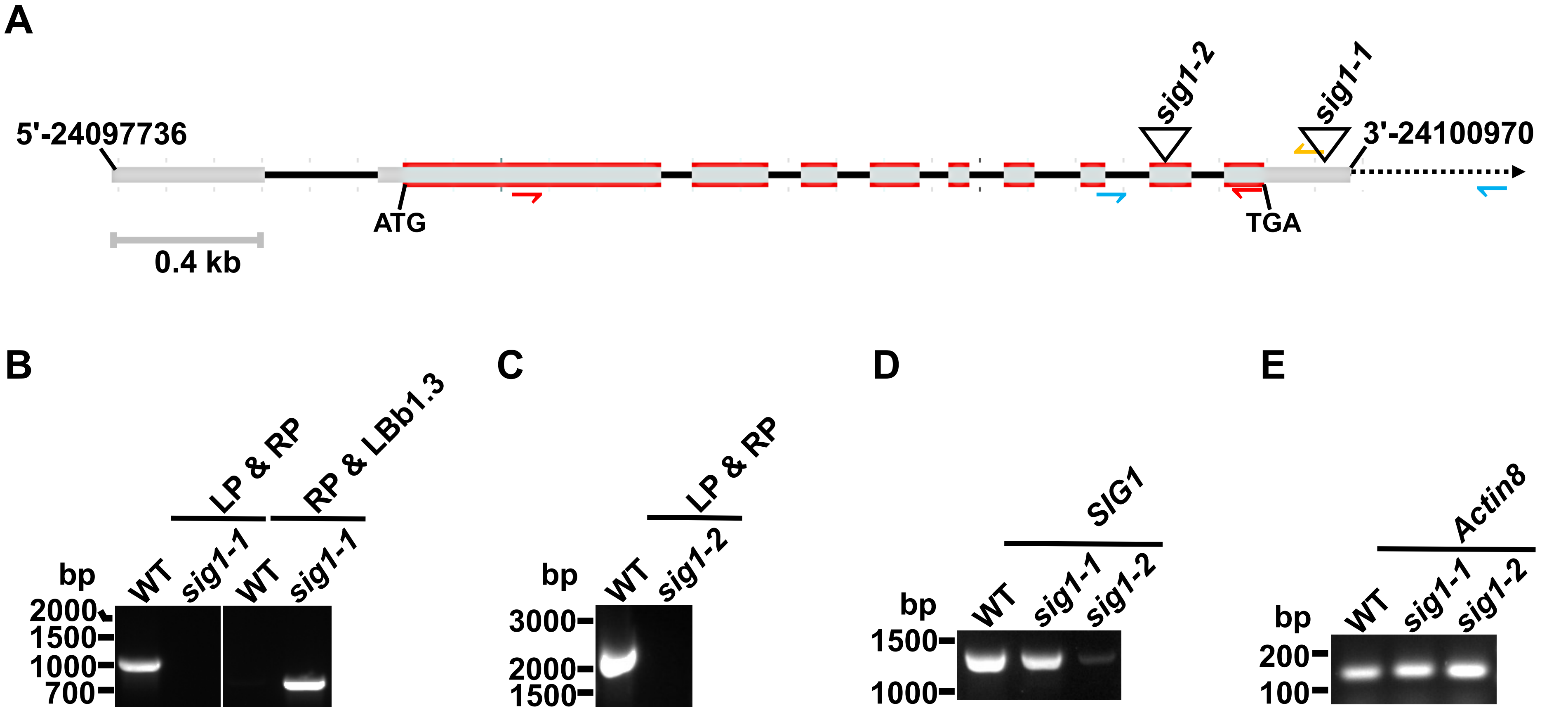

Supplementary Fig. 1. Genotyping of *SIG1* T-DNA lines. (A) A scaled model of the *SIG1* exon-intron regions. Gray rectangles represent exons and the thick black connecting lines stand for introns. Red lines demarcate the coding regions of the exons. The dotted black line denotes genomic region downstream of *SIG1*. SIG1's 5' to 3' genomic coordinates, and start (ATG) and stop (TGA) codons are indicated. The T-DNA insertion sites of *sig1-1* (SALK_147985c) and *sig1-2* (CS371990) are shown by downward-pointing triangles. (B) A genomic PCR with LP and RP primers (blue half arrows) gives the expected 1070 bp fragment in wild type but not in *sig1-1,* consistent with a T-DNA insert in the mutant. A further genomic PCR with RP and T-DNA left border (LBb1.3) primers (blue and orange arrows, respectively) shows the predicted ~700 bp T-DNA fragment in *sig1-1* (C) A genotyping PCR with genomic primers (red half arrows) amplifies a 2010 bp fragment in wild type but not in *sig1-2*, in line with a T-DNA insert in the latter. (D) A genotyping RT-PCR amplifying the coding sequence of mature SIG1 gives a 1269 bp fragment in wild type, *sig1-1* and *sig1-2*. Compared to wild type, the band intensity is however much lower in mutants, consistent with the knockdown effect of the T-DNA mutagen on *SIG1* transcription. The *sig1-2* genomic PCR (C) and *SIG1* RT-PCR (D) share the same primer pair (red half arrows). (E) An *Actin8* control RT-PCR shows similar band intensity in all three genotypes, confirming the integrity of the RNA template.

A

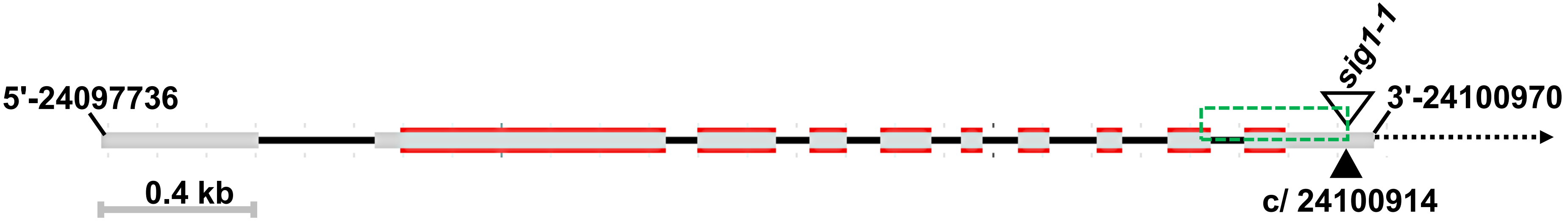

AGCGTGGACCGCTTGCTGCAACTCTCTCAGGGCCAGGCGGTGAAGGGCAATCAGCTGTTGCCCGTCTCACTGGTGAAAAGAAA
AACCACCCCAGTACATTAAAAACGTCCGCAATGTGTTATTAAGTTGTCTAAGCGCCATATTACATAACTGTCTCTTGTTTCTCAACTC
AGTTTACGAAAGAAGCAAGGAAGATAAAAAAATAAAAGAGAGTAGTACTCTTCCCCCTTTTTTGTCCTGTATATATACACATTCACAA
CCGCTCTCTATGGCTCTGATGTAATCAAAATGTACAAAAACAAAATCAATTCTTAAGGATCATTGCCTCCATTTTTCTCTTCCTCGCT
GCGTGTTTTAGTTTCTCCAGTGCCACAAGCCCTACCTGTCTCACCCTCTCTCTCGATAATCCTATCCTACAATTTTTCAAGAGACAG
TTTAAAGAATGATGTTGATGATGCAACAATGGACCAAAAGGCTTAAGATTGGATACATACCGTTTACTAATGTCTTCCCATGTGAGA
CATTCTTTGTCTAGACCATAGTATAATCGAATGATCTCTTTTTCCCGTTCTCCGAGTGTTGCACTTATAAGCTTGCTTACTTCCTCCT
GCAATTTTATCATCAATCTCATATGCTCAAACTCTAGTGTTTCATGATAGAGAAAATCAGAACCATTGTATTAAATGTAGCTTCACTTA
TGACTTGTTCTTTTTTTTTTTTTNACCTTGA

B

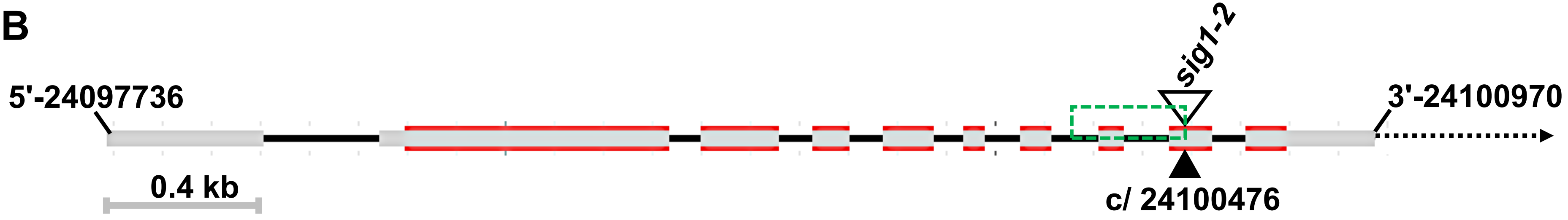

TTACAAGAGTATTGCACTTATAAGCTTGCTTACTTCCTCCTGCAATTTTATCATCAATCTCATATGCTCAAACTCTAGTGTTTCATGAT
AGAGAAAATCAGAACCATTGTATTAAATGTAGCTTCACTTATGACTTGTTCTTTTTTTTTTTTTTACCTTGAGGGCCAAATCATCATACC
CGTGCCACGGATTGTTCTCCAAACGAGTATCCCCAATGTACTGGAAATAGTGAAACAAACAAGCAAATCGTTATAAACTGGAAAGG
AAAAACCCATGGAAACTTGGATGGGGAAAACAAAATCGTCGTTTTGTTTTAAAAAAAACCGAAAAAAAAACTCCCACTGTGATGAG
TTTCTCCAGGGAGACCATTCAAAAAAGGAAATGCATCTCTGTCTAAAAAAAATACTTTGCTTACACCCTGTATTTTGCAAATGCAAA
AAAGTAAGCACAAATACACATAGATCCCC

Supplementary Fig. 2. T-DNA insertion sites of *sig1* mutants as confirmed by sequencing. (A) T-DNA insertion site in *sig1-1.* Sequencing of the T-DNA amplificate (Supplementary Fig. 1B) using the T-DNA left border primer (LBb1.3) identifies the T-DNA-genomic junction in *sig1-1*. Sequences that match *SIG1* gene are colored in blue and the T-DNA sequence in red. (B) T-DNA insertion site in *sig1-2*. Confirmation sequence as supplied by the GABI-Kat database. Sequences that match *SIG1* are in blue.

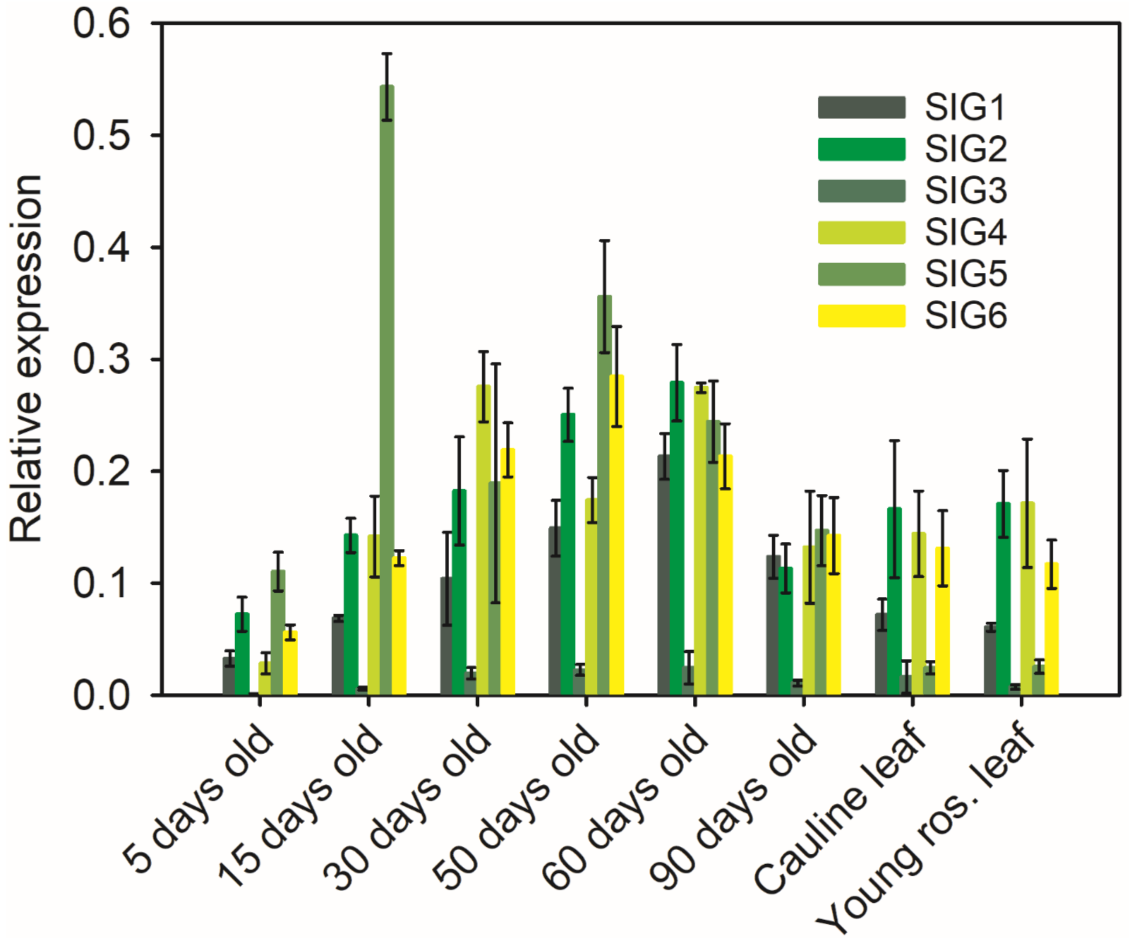

Supplementary Fig. 3. A developmental profile of sigma factor gene expression. Age-dependent transcript accumulation of sigma factor genes in *Arabidopsis* leaves as analyzed by qPCR. Expression values have been normalized to the *Actin8* housekeeping gene. “Young ros. leaf” stands for young leaves of vegetative rosette. Error bars represent ±SE of the mean of three biological replicates.

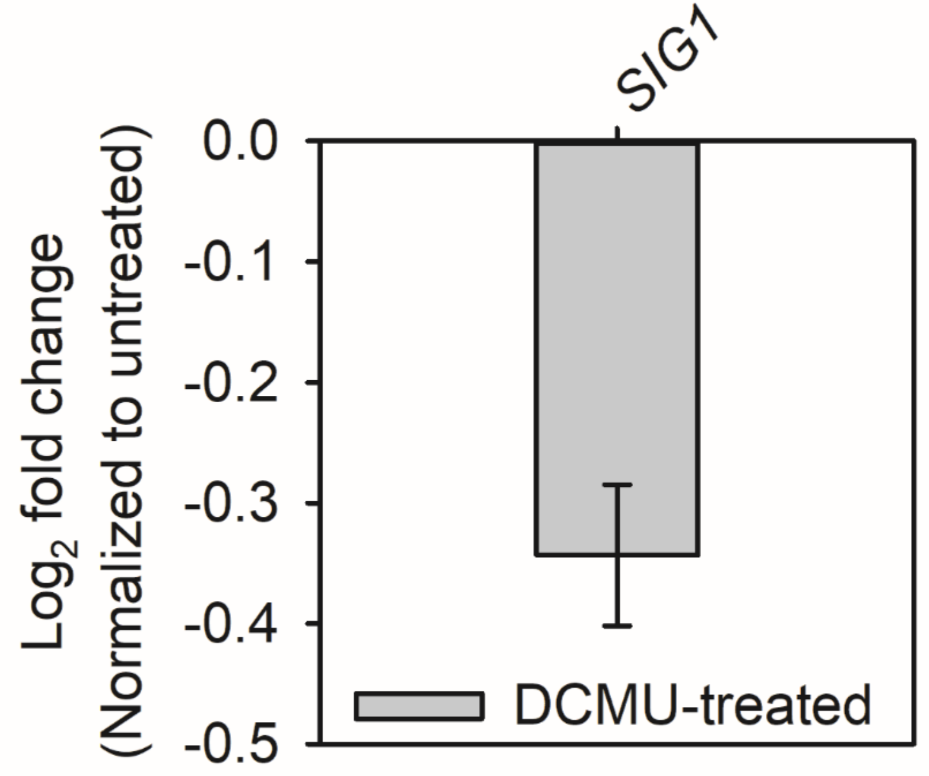

Supplementary Fig. 4. DCMU treatment decreases *SIG1* transcript accumulation. The *SIG1* transcript abundance in DCMU-treated sample as analyzed by qPCR. The log2 fold change after normalization with the untreated sample is shown. Error bars represent ±SE of the mean of four biological replicates.

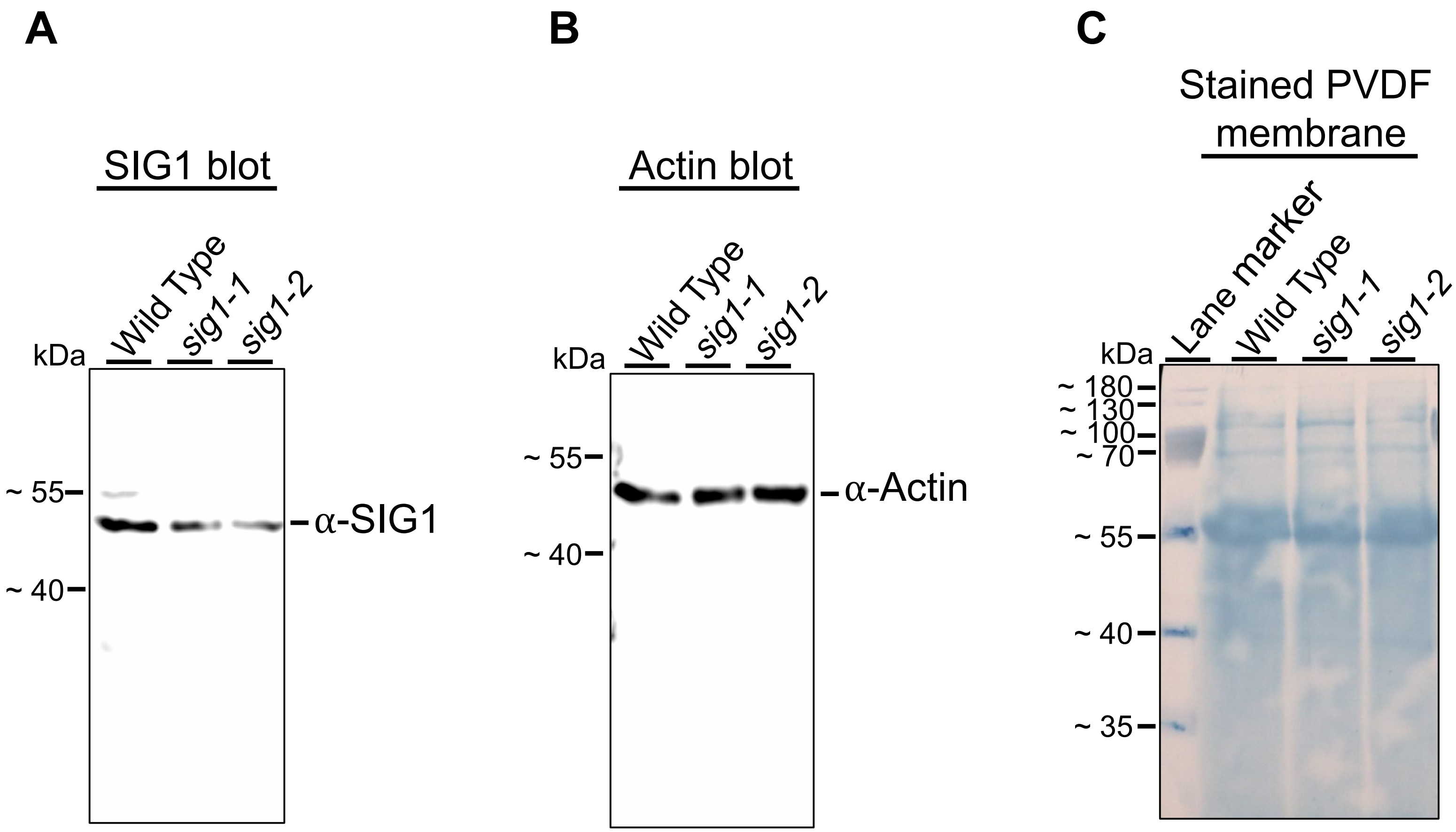

Supplementary Fig. 5. The full uncropped western blots of SIG1 (A), Actin (B) and the corresponding stained PVDF membrane (C) presented in Fig. 1B.
