# Supplementary material for: Sigma factor 1 in chloroplast gene transcription and photosynthetic light acclimation
Source: J Exp Bot. 2019 Oct 23;71(3):1029–38. doi: 10.1093/jxb/erz464 (PMC6977190; doi:10.1093/jxb/erz464)
Supplement: erz464_suppl_Supplementary_Table_S1 [file erz464_suppl_supplementary_table_s1.pdf]

Supplementary Table 1. Primers used for qRT-PCR, genotyping and complementation

Purpose	Gene	Primer name	Sequence 5' to 3'
qRT-PCR	*PsaA*	PsaA-F	GGCACAAGCATCTCAGGTAA
		PsaA-R	AGCCCAAACAATGGATTCAA
	PsbA	PsbA-F	GGTTACAGATTCGGGCAAGA
		PsbA-R	AATACCTACTACCGGCCAAGC
	PsbB	PsbB-F	CTGCTCTAGTTGCTGGTTGG
		PsbB-R	CGCCCCAAGAATTGGTTATT
	PsbD	PsbD-F	CTTTAGGGGGTTGGTTCACA
		PsbD-R	GCTTCAGGACCCCACAGTAA
	PsbE	PsbE-F	TGTCTGGAAGCACAGGAGAA
		PsbE-R	GAGGGCTCCCGAACACAT
	PetB	PetB-F	ATTGGGCGGTCAAAATTGTA
		PetB-R	AGACGGCCGTAAGAAGAGGT
	AtpB	AtpB-F	ATGAGTGCGACAGAGGGTTT
		AtpB-R	TGTGCGAGTATCGACAGGAC
	NdhB	NdhB-F	TACGAAGGATCTCCCACTCC
		NdhB-R	GCTAAGAATAGCTAGGATTTCCAGA
	RbcL	RbcL-F	CACCACAAACAGAGACTAAAGCA
		RbcL-R	CCTGCTTCTTCAGGTGGAAC
	TrnD	TrnD-F	GGGATTGTAGTTCAATTGGTCAGA
		TrnD-R	GACTGACGGGGCTCGAA
	RpoB	RpoB-F	TGCTAAGTTTCCAAAAATTGAAGA
		RpoB-R	CAAATTAATCCCGCGGATAC
	RpoTp	RpoTp-F	GAGCAGGAGGTGAGGATTCA
		RpoTp-R	ACACGTTTCCTAAGCAATTGC
	Lhcb1.2	Lhcb1.2-F	CATCTGAAGTCCTCGGAAGC
		Lhcb1.2-R	GGAACTCTCCGGTAAGGTAGC
	Lhcb2.2	Lhcb2.2-F	CTCCCCAAAGCATCTGGTAT
		Lhcb2.2-R	GCTCACGGTTCTTAGCGAAT
	SIG1	SIG1-F	TCATAAGTCAAGATTGAAGGATAGATT
		SIG1-R	CAAACGCACATTACTCATAGCC
	SIG2	SIG2-F	TGTGCCCCTAAACACAACAA
		SIG2-R	TGCCTGTCTGATATAAGGGAATC
	SIG3	SIG3-F	TCCCAAACAGATTGTTGGTTC
		SIG3-R	CAATAACGAGAGCCAATGTTTA
	SIG4	SIG4-F	TTGTACCTCAAGGAAGGAGCA
		SIG4-R	CTTTTCTCTCGCTTCTTTTCTACG
	SIG5	SIG5-F	TGAAGGCTCTTCTTGAAGTGAA
		SIG5-R	TTAATAAGTTTGTTTCTTGCAGCTC
	SIG6	SIG6-F	TCCTTCCTCTGTAGTGATGCTT
		SIG6-R	TCGTCTCTTAACGCATGTGA
	Actin8	Actin8-F	TTCCAGCAGATGTGGATCTCTA
		Actin8-R	AGAAAGAAATGTGATCCCGTCA
Genotyping	*SIG1* (SALK_147985c)	985-LP	TGTATTCCTGAAACCGGAGTG
		985-RP	ATGATGATTTGGCACTCAAGG
	SIG1 (CS371990)	990-LP	GCTTCTACTGAGAAGCCA
		990-RP	TCAATTCTTAAGGATCAT
	LBb1.3	LBb1.3	ATTTTGCCGATTTCGGAAC
	SIG1 (RT-PCR)	SIG1-F	GCTTCTACTGAGAAGCCA
		SIG1-R	TCAATTCTTAAGGATCAT

	Actin8 (RT-PCR)	Actin8-F	TTCCAGCAGATGTGGATCTCTA
		Actin8-R	AGAAAGAAATGTGATCCCGTCA
**Complementation**	*SIG1* (SALK_147985c)	C-XhoI-F	GCGCGCctcgagATGGCTACTGCAGCTGTT
		C-EcoR1-R	GCGCGCgaattcTCAAGCGTAATCTGGAACATCGTATGGGTAATTCTTAAGGATCATTGC

Letters in lowercase denote restriction sites. The stop codon is shaded in red and the coding sequence for the HA epitope is highlighted in green.
